# Inhibition of snake venom induced sterile inflammation and PLA2 activity by Titanium dioxide Nanoparticles in experimental animals

**DOI:** 10.1038/s41598-019-47557-y

**Published:** 2019-08-01

**Authors:** Shubhro Chakrabartty, Md. Iqbal Alam, Saumya Bhagat, Aftab Alam, Neha Dhyani, Gausal A. Khan, M. Sarwar Alam

**Affiliations:** 1 Department of Electronics and Communication Engineering, NIT, Goa, India; 2 Department of Physiology, Hamdard Institute of Medical Sciences & Research, Jamia Hamdard, New Delhi, India; 3 Hematology division, Defence Institute of Physiology and Allied Sciences, DRDO, Delhi, India; 4 Division of Neurosurgery, Department of Clinical Neurosciences, University of Cambridge, Cambridge, UK; 5 Department of Chemistry, School of Chemical and Life Sciences, Jamia Hamdard, New Delhi, India

**Keywords:** Nanobiotechnology, Biomarkers

## Abstract

Sterile inflammation (SI) is an essential process in response to snakebite and injury. The venom induced pathophysiological response to sterile inflammation results into many harmful and deleterious effects that ultimately leads to death. The available treatment for snakebite is antiserum which does not provide enough protection against venom-induced pathophysiological changes like haemorrhage, necrosis, nephrotoxicity and often develop hypersensitive reactions. In order to overcome these hindrances, scientists around the globe are searching for an alternative therapy to provide better treatment to the snake envenomation patients. In the present study TiO_2_ (Titanium dioxide)-NPs (Nanoparticles) has been assessed for antisnake venom activity and its potential to be used as an antidote. In this study, the synthesis of TiO_2_-NPs arrays has been demonstrated on p-type Silicon Si < 100 > substrate (∼30 ohm-cm) and the surface topography has been detected by Field-emission scanning electron microscopy (FESEM). The TiO_2_-NPs successfully neutralized the *Daboia russelii* venom (DRV) and *Naja kaouthia* venom (NKV)-induced lethal activity. Viper venom induced haemorrhagic, coagulant and anticoagulant activities were effectively neutralized both in *in-vitro* and *in vivo* studies. The cobra and viper venoms-induced sterile inflammatory molecules (IL-6, HMGB1, HSP70, HSP90, S100B and vWF) were effectively neutralised by the TiO_2_-NPs in experimental animals.

## Introduction

Biomedical therapy and diagnostics are getting more prominent with the utilization of nanotechnology concept^1^. The aim of using a nano-medicine is to improve the bio-distribution of therapeutic agent and to allow its accumulation on the target site^2^. Different types of nano-medicine have been evaluated in recent years, f.e., liposomes, polymers, micelles and antibodies. Several evidences also indicate the ability of these nano-sized carrier materials to improve the balance between the efficacy and the toxicity of therapeutic interventions.

Today, nano-scale research has been emerged as important players in the field of pharmacology and biotechnology. Except few nanoparticles and quantum dots, most of them are toxic in nature and are not applicable for medical or therapeutic applications^3^. TiO_2_ has a wide spectrum of properties that varies with different techniques used for its synthesis^4^ and has applications in the field of biomedical research and treatment such as anti-tumour therapy^5,6^, manufacturing of bio-products^7^ etc. Ubiquitous applications and better results of TiO_2_-NPs, makes them a potential candidate for biomedical research^8^. In the present study, the synthesis of TiO_2_-NPs arrays has been demonstrated on p-type Silicon Si < 100 > substrate (∼30 ohm-cm) and the surface topography has been detected by Field-emission scanning electron microscopy (FESEM). The size of the deposited TiO_2_-NPs is maximum in the range of 5–6 nm. The Energy-Dispersive X-ray (EDX) mapping of FESEM image showed the presence of Titanium (Ti), Oxygen (O_2_) and Si (Substrate) in the sample. Raman analysis confirms the crystalline nature of TiO_2_-NPs with Anatase and Rutile modes.

Present day lifestyle increases the risk of several deadly diseases including cardiovascular disorder, diabetes, cancer, etc. In these cases early detection and treatment can decrease the mortality rates. Apart from these lifestyle related diseases, some clinical conditions such as snake envenomation also pose a major public health challenge across the world. About 5.4 million annual snakebite cases are reported globally and more than 100,000 people die each year as a result of snakebites of which more than 50,000 deaths occur in India alone^9^. Majority of deaths reported are particularly from rural and agriculturally active areas. WHO has also declared snakebite as a ‘neglected tropical disease’ and is now a global health priority^9–12^. Antiserum is the only choice to treat snakebite patients, which again is not always effective against venom-induced haemorrhage, necrosis, nephrotoxicity and often develop hypersensitive reaction^13–17^. Antiserum development in animal is time consuming and needs cold chain management. Therefore, there is a need to develop suitable therapeutic agent for the treatment of snakebite. Now-a-days use of nanoparticles are quite popular and frequently used as a drug-delivery vehicles^2,18^. TiO_2_ is one such promising material because of its wide spectrum properties that varies with different techniques used for its synthesis^3^ and has been reported for various applications in the field of biomedical research^4–6^.

Sterile inflammation is an essential process in response to snakebite and injury. The pathophysiological response to sterile inflammation results into many harmful and deleterious effects that ultimately lead to death. The harmful effects of snake envenomation are haemorrhage, necrosis, renal failure, cardiotoxicity, neurotoxicity, myotoxicity and hematoxicity. These deleterious effects could be due to the increased formation of sterile molecules: HMGB1, HSPs, S100B and IL6. HMGB1 which has recently been drawn much attention because of its binding ability to cell signalling receptors like RAGE, TLR2, TLR4 and TLR9 receptors. These receptors have major role in the initiation of inflammatory response and they also participate in development and progression of haemorrhage, necrosis, and cell death. Similar pathophysiological changes were also observed in case of snake envenomation. Therefore, in this paper we have shown the expression of the sterile inflammatory marker molecules developed in response to snakebite in experimental animals and its neutralization by TiO_2_.

## Materials

### Chemicals

All chemical used in this study were of analytical grade. The solvents used were prepared in distilled water.Anti-Hsp70 antibody, Anti-Hsp90 antibody, Anti-IL-6 antibody, Anti-Von Willebrand Factor antibody, Anti-S100 beta antibody, Anti-HMGB1 antibody-ChIP Grade were purchased from Abcam (Cambridge, MA, USA); and O-Phenylenediamine dihydrochloride and bovine serum albumin were purchased from Sigma Aldrich (St. Louis, MO, USA). MaxiSorp flat-bottom 96-well plates were purchased from Nunc GmbH & Co, Langenselbold, Germany.

## Methods

### Animals

Male Swiss albino mice (18–20 g) used in this study was obtained from the animal house of Jamia Hamdard (Hamdard University), New Delhi. Guidelines of the Institutional Animal Ethical Committee has been followed for the care and handling of animals. All mice were housed five/cages and fed standard laboratory diet and water *ad libitum* with 12-h dark/light cycles at constant temperature of 25 ± 2 °C. ‘Principles of laboratory animal care’ (NIH publication No. 85-23, revised in 1985) as well as Indian laws on ‘Protection of Animals’ have been followed for all the animal experiments under the provision of authorized investigators. Experiments involving the use of mice were approved by the Committee for the Purpose of Control and Supervision of Experiments on Animals (CPCSEA) of the Jamia Hamdard (Deemed University), New Delhi (permission #173/GO/Re/S/2000/CPCSEA). Experiments involving animals meet the International Guiding Principles for Biomedical Research Involving Animals (CIOMS).

### Preparation of nanoparticles

The GLAD technique was carried out to deposit TiO_2_ (High purity) (99.99%, MTI, USA) NPs of 5 nm range inside the electron beam chamber (Hind High Vacuum Co. (p) Ltd., 15F6) on p-type Silicon Si <100> substrate (∼30 ohm-cm) at a base pressure of ~2 × 10^−5^ mbar^19^. The substrates were rotated azimuthally with constant speed of 460 rpm at an orientation of 85° with respect to the perpendicular line between the metal source and the planar substrate holder^20^. A low deposition rate of 1.2 Å/s was kept constant, which was monitored by a quartz crystal. Then the grown TiO_2_-NPs were ultrasonicated in electron grade acetone to make a liquid solution. The surface morphology of randomly placed TiO_2_-NPs over n-type Si substrate of probably 5 nm was observed using an Atomic Force Microscope (BrukerInnova).

### Preparation of venoms

Lyophilized snake venom from *Daboia russelii* (Viper), and *Naja kaouthia* (Cobra) were obtained from Calcutta Snake Park (Kolkata, India) and was preserved in desiccators at 4 °C in an amber-coloured glass vial until further use. The snake venom was dissolved in 0.9% saline and centrifuged at 2000 rpm for 10 min. The supernatant was used as venom and kept at 4 °C and used within three month. The venom concentration was expressed in terms of dry weight (mg/ml, stock venom solution)^21^.

### Venom inhibiting activity

To determine the venom inhibiting activity of the nanoparticles, following pharmacological experiments were performed:

#### Inhibition of venom lethal effect

Snake venom toxicity assessment was done by intravenous (*i.v*) injection of venom prepared in 0.2 mL physiological saline at different concentration in male albino mice weighing18–20 g^22,23^. Assessment of *in vitro* antagonism was done by mixing different concentrations of venom (2.2–22 µg) with a fixed amount of TiO_2_-NPs, the mixture incubated at 37 °C for 1 hour, and then centrifuged at 2000 rpm for 10 minutes. The supernatant was injected intravenously (*i.v*) into male albino mice, six mice per dose. The median lethal dose (MLD_50_) was calculated 24 hours after injection of the venom-TiO_2_-NPs mixture. Lethal toxicity was also assessed by subcutaneous (*s.c)* injection of various doses of venom. The neutralizing potency of TiO_2_-NPs was assessed by injection (*s.c*) of venom (45–225 µg) into groups of six mice followed by immediate injection of fixed dose of TiO_2_-NPs (5 ng) intravenously^24^.

#### Inhibition of venom haemorrhagic activity

The minimum haemorrhagic dose (MHD) of venom which when administered into mice causes development of haemorrhagic lesion of 10 mm diameter within 24 hours^23^. This lesion was measured and the estimation of neutralization of the haemorrhagic activity was done by mixing a fixed amount of TiO_2_-NPs (2 ng) with different amounts of venom (5–25 µg). The mixture of TiO_2_-NPs- venom was incubated at 37 °C for 1 hour, then spin at 2000 rpm for 10 min and finally 0.1 ml of supernatant taken were injected intradermally (*i.d*)^25^. After 24 hours haemorrhagic lesion was estimated. To assess the anti-haemorrhagic activity of venom *in vivo*, various amount of venom (5–15 µg) were injected (*i.d*) followed by the TiO_2_-NPs (5 ng, intravenously) and the haemorrhagic lesion measured after 24 hours.

#### Inhibition of venom necrotic activity

To assess the *in vitro* anti-necrosis effect of TiO_2_-NPs, various concentration of venom (5–25 µg) were incubated with fixed amount of TiO_2_-NPs and administered intradermally (*i.d*) into mice. The necrotic lesion was estimated after 48 hours. In *in vivo* study, the venom (*i.d*) (5–15 µg) was injected followed by injection (*i.v*) of TiO_2_-NPs and observed after 48 hours.

#### Inhibition of venom defibrinogenating activity

Defibrinogenating activity of venoms or toxins is expressed as the minimum defibrinogenating dose (MDD). MDD of DRV is defined as the minimum amount of venom which when injected (intravenously) into mice causes incoagulable blood 1 hour later. Neutralization of this activity was estimated by mixing different amount of venom (2.5–12.5 µg) with fixed amounts of TiO_2_-NPs, incubating at 37 °C for 1 hour and centrifugation. The supernatant was injected (*i.v*) into albino mice (18–20 g) as described above (*in vitro*). For *in vivo* studies, the MDD of venom was injected (*i.v*) followed by the TiO_2_-NPs (*i.v*) and the nature of the blood observed after 1 hour^26^.

#### Inhibition of venom PLA_2_ effect

For carrying out the PLA_2_ inhibition activity, 2 ml of egg yolk suspension, 0.2 ml of test material (venom, TiO_2_-NPs and Venom + TiO_2_-NPs) were mixed in the different test tube and kept for 1 hour incubation at 37 °C. The test tube containing test materials were kept on a water bath and the time required for coagulation was recorded. A blank was run with normal saline in place of test material. One unit enzyme activity was defined as the amount of venom, which increased the coagulation time of the egg yolk control by one minute^27^. Estimation of neutralization of the enzyme activity, was done when fixed amount of TiO_2_-NPs (2 ng) were mixed with different amount of viper venom (2–10 μg) and the mixture was incubated for 1 hour at 37 °C. Centrifuged at 2000 rpm for 10 minutes, supernatant was tested in a total of 0.2 ml for the enzyme neutralization activity.

#### Inhibition of venom-induced mouse paw oedema

The minimum oedematic dose (MOD) of venom/carrageenan is defined as the least amount of venom/carrageenan which when injected into male albino mice produced inflammation (oedema) in the paw. Non-fasted male albino mice (18–20 g) were treated with different doses of venom, carrageenan and TiO_2_-NPs. To assess the anti-inflammatory activity of TiO_2_-NPs various amount of venom/carrageenan (in 0.01 ml) were injected (intraplantar) followed by the intraperitoneal (*i.p*) injection of TiO_2_-NPs (250 ng/kg) and anti-inflammatory activity was measured. For control, equal amounts of saline were injected (intraplantar). The oedematogenic response was evaluated by the use of a screw gauge at given time intervals. Oedema was reported as the percentage difference between the values obtained from the injected paw with the carrageenan, venom, venom + TiO_2_-NPs, carrageenan + TiO_2_-NPs and saline, as described by Trebien and Calixto^24,28^.

#### Inhibition of Sterile inflammatory Markers

Different pro-inflammatory markers such as heat shock proteins (HSP70 and HSP90), high mobility group box 1 (HMGB1), interleukin 6 (IL-6), von Willbrand factor(vWF), and S100 calcium-binding protein B (S100B) was done by ELISA as described by Engvall *et al*.^29^. Briefly, 50 μg of proteins were used with bicarbonate coating buffer to coat the wells in 96-well plates and incubated over night at 4 °C. Nonspecific binding was blocked with 5% BSA and then the samples were washed with PBST (0.05% Tween-20) and incubated for 3 hours with respective primary antibodies (against Hsp70; Hsp90; HMGB1); IL-6; vWF; S100b (1:1000). Next, the wells were washed and incubated with HRP conjugated secondary antibodies for 1 hour, and then washed with PBST and incubated with OPD substrate solution. The reaction was terminated by adding H_2_SO_4_ (1 N) and absorbance was measured at 450 nm. Using similar methods, a standard ELISA curve for absorbance versus specific antigen concentration was constructed using respective recombinant proteins.

## Results

### Topographic image of TiO_2_-NPs

**Figure 1:**
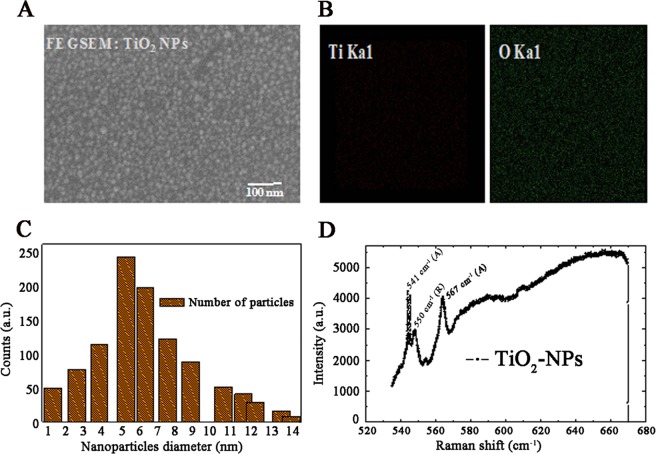
Structural behaviour of TiO_2_-NPs. (**A**) Top view FESEM images of nano-sized TiO_2_ particles. (**B**) Element mapping of the sample. (**C**) Particle histogram of Image 1(**A**). (**D**) Raman spectrum of TiO_2_ -NPs.

In case of GLAD, the TiO_2_-NPs are formed due to the atomic shadowing effect on the arbitrarily deposited seeds. The diffusivity of the landed atoms on the substrate depends on the rate of evaporation. A lower evaporation rate of the material will enable the landed atoms to diffuse for longer distances before they get impinged by next arriving atoms. This can lead to an increase in the capture radius of the deposited atoms and hence the atoms nucleation area increases. Figure 1A shows a top view of grown TiO_2_-NPs on p-type Si substrate prepared at 85° and 460 rpm azimuthal rotation to the sample, the images were captured by FESEM, keeping the gun vacuum on 2.18 e-009 mBar. The EDX element mapping reveals Titanium (Ti, brown color) and Oxygen (O, green color) are present in the sample shown in Fig. 1B. Figure 1C projects the particle counts histogram of FEG: SEM image 1 A, the grown particles were in the range of 1–14 nm, where maximum number of NPs had a count of 5–6 nm. The Raman spectra processing of TiO_2_ samples shows that the grown nanoparticles are having two Anatase and Rutile modes^30^ (Fig. 1D).

### Raman analysis

Raman spectroscopy is a flexible and most authenticated technique used for ubiquitous sectors. It is a very prominent tool for nano-biomedical studies and also helpful in the analysis of food and pharmaceutical non-materials. It has a great impact on drug analysis due to its simplicity in minimal sample handling and the significant differences in scattering strength between packaging materials tablet excipients, and active drug components^31^. Figure 1D shows the dependence of the Raman scattering intensity on the incident laser wavelength (laser excitation 532 nm). We have noticed that our prepared TiO_2_-NPs have crystalline nature and also have some characteristic peaks.

### Properties of snake venoms and TiO_2_-NPs

LD_50_ of *DRV* was found to be 2.2 μg intravenously and 45 μg when injected subcutaneously in mice (18–20 g); whereas *NKV* was 2.8 μg intravenously and 4.61 μg subcutaneously. The MHD/MND was 5 μg per animal (18–20 g) of *DRV*. MDD was 2.5 μg per animal (18–20 g) of *DRV*. The concentration of viper and cobra venoms in terms of protein were found to be 91.5% and 95% respectively. The TiO_2_-NPs [up to 25 ng per animal (18–20 g); *i.v*] did not produce any lethal effect up to 48 hours of observations^22^.

### Lethal activity

In *in-vitro* study, viper and cobra venoms (1–10 LD_50_) were incubated with TiO_2_ -NPs (2 ng) and gave protection against venom-induced lethality. In *in vivo* study, cobra venom (1–5 LD_50_) was injected (*s.c*) into male albino mice followed by TiO_2_-NPs (5 ng/mouse, *i.v*). Viper and Cobra venom-induced lethality was significantly antagonized by TiO_2_-NPs as compared with the control animal (venom only) (Table 1). The ED_50_ of the TiO_2_-NPs was observed as 2 ng *in vitro* and 4 ng in *in vivo* respectively against viper venom (Table 1).

**Table 1:** Inhibition of lethal action of Viper and Cobra venom by TiO_2_-NPs. Results are expressed as mean of six observations. **Duration of deaths time following venom exposure (venom control animal): DRV (1 Median Lethal Dose) = 13.24 ± 0.30 hours; Cobra (*Naja kaouthia*) venom (1 Median Lethal Dose) = 6.20 ± 0.30 hours.

TiO_2_ (ng)	Viper Venom (μg)	Fold of neutralization (in terms of LD_50_)	Cobra Venom (μg)	Fold of neutralization (in terms of LD_50_)
**Venom nano-particles incubated 37** **°C**, **60 min and injected i**.**v**				
TiO_2_ (2)	11	5	6	2.1
Control				
**Venom injected (s**.c) ** followed immediately by TiO_**2**_ **(i**.**v)**				
TiO_2_ (5)	45	1.0	10	2.1

### Haemorrhagic and necrotic activity

In *in-vitro* study, Viper venom (20 µg) incubated with TiO_2_ (2 ng) and injected 0.1 ml (*i.d*) into mice. It showed protection against venom induced haemorrhagic activity (Table 2). In *in-vivo* study, venom (10 µg) injected (*i.d*) into mice followed by the injection (*i.v*) of TiO_2_-NPs (5 ng) gave protection against venom-induced haemorrhagic activity. The degree of protection in *in-vivo* was less than that of *in-vitro* studies.

**Table 2:** Inhibition of haemorrhagic action of Viper venom by TiO_2_-NPs.

TiO_2_ (ng)	Viper Venom μg)	Fold of neutralization (In terms of MHD)
**Venom**-**TiO**_**2**_ **nanoparticles incubated 37** **°C**, **60 min and injected i**.**d**		
TiO_2_ (2)	20	4
**Venom injected (i**.**d) followed immediately by TiO**_**2**_ **(i**.**v)**		
TiO_2_ (5)	10	2

*Results are expressed as mean of six observations. *i.d* = intra-dermal. *i.v* = intra-venous.

### Defibrinogenation activity

The TiO_2_-NPs effectively antagonized the viper venom-induced defibrinogenating activity. In *in-vitro* study, the TiO_2_-NPs (2 ng) gave protection up to 2 MDD (5 µg) against venom-induced defibrinogenation. In *in-vivo* study, venom-induced defibrinogenation was antagonised by TiO_2_-NPs. The fold of protection was always higher in *in vitro* studies (Table 3).

**Table 3:** Inhibition of defibrinogenating activity of Viper venom by TiO_2_-NPs.

TiO_2_ (ng)	Viper Venom (μg)	Fold of neutralization (In terms of MDD)
**Venom-nanoparticles incubated 37** **°C**, **60 min**. **and injected (i**.**v)**		
TiO_2_ (2)	5.0	2.0
**Venom injected (i**.**v) followed immediately by TiO**_**2**_		
TiO_2_ (5)	3.5	1.5

*Results are expressed as mean of 6 observations. MDD = Minimum Defibrinogenating Dose = 2.5 μg.

### Neutralisation of Venom Phospholipase A_2_ (PLA_2_) activity

Egg yolk coagulation method was performed to assess the PLA_2_ activity of viper venom. 1 unit of venom activity which increased the coagulation time by 1 minute was found to be 2 μg (control 0.9% saline, coagulation time was found to be 45 ± 1.16 seconds). The TiO_2_-NPs were tested for Phospholipase A_2_ activity by incubating with different amount of venom (10 μg). The venom PLA_2_ was effectively neutralized by the TiO_2_-NPs (Table 4).

**Table 4:** Inhibition of Phospholipase A_2_ activity of Viper venom by TiO_2_-NPs.

Group	Viper Venom (μg)	Fold of neutralization (In terms of unit)
**Control (only Venom)**		
Viper (2 µg)	2(1)	1
**Venom-NPs incubated 37** **°C**, **60 min then added to egg yolk suspension**		
TiO_2_ (2 ng)	10	5

Results are expressed as mean of ten observations. 1 unit = one unit of enzyme activity of DRV was defined as the amount of venom which increased the coagulation time of egg yolk by one minute.

### Mouse paw oedema

The assessment of anti-inflammatory activity of the TiO_2_-NPs was done by mouse paw oedema. Mouse paw oedema was induced by venom, attained its peak at 1 hour of observation. The TiO_2_-NPs at a dose of 5 ng/mouse (*i.p*) was found to produce significant inhibition of venom-induced inflammation. Inhibition of inflammation induced by TiO_2_-NPs was maximum (51.3 ± 2.5%) at 2 hours of observation, as compared with aspirin (42.8 ± 1.2%) and indomethacin (40.3 ± 1.3%). Carrageenan (300 µg in 0.01 ml) injection produced significant inflammation in mouse paws. Pre-treatment with TiO_2_-NPs (5 mg/kg, *i.p*) before carrageenan injection produced a significant reduction in oedema (Table 5).

**Table 5:** Viper venom induced inflammation and inhibition by TiO_2_-NPs.

Groups	Venom/Carrageenan µg (MED)	Oedema (%)			
		1 hour	2 hour	3 hour	4 hour
Control	(0.9% saline)				
Viper venom	001(1)	105.3 ± 5.8	87.5 ± 07.7	83.74 ± 5.9	78.43 ± 6.5
Carrageenan	300(1)	054.4 ± 7.2	47.9 ± 11.3	35.20 ± 7.6	34.60 ± 5.3
**Inhibition studies (Carrageenan injected 300(1) into intraplantar surface followed immediately by TiO**_**2**_**-NPs)**					
TiO_2_-NPs (250 ng/kg)	5(5)	47.5 ± 1.7	51.3 ± 2.5	52.0 ± 1.2	52.8 ± 2.9
Aspirin (10 mg/kg)	5(5)	43.9 ± 0.9	42.8 ± 1.2	42.2 ± 1.7	42.1 ± 0.8
Indomethacin (10 mg/kg)	5(5)	40.8 ± 1.9	40.3 ± 1.3	40.1 ± 0.5	39.9 ± 2.1
**Inhibition studies (Carrageenan injected 300(1) intraplantar surface followed immediately by TiO**_**2**_**-NPs)**					
TiO_2_-NPs (250 ng/kg)	600(2)	46.0 ± 1.8	49.6 ± 2.4	48.2 ± 1.6	48.0 ± 3.2
Aspirin (10 mg/kg)	600(2)	37.6 ± 2.7	42.6 ± 3.2	39.7 ± 2.7	40.1 ± 3.1
Indomethacin (10 mg/kg)	600(2)	35.1 ± 1.5	35.7 ± 2.8	35.3 ± 2.5	35.0 ± 1.4

Venom and carrageenan were injected intraplantar in the foot pads. Drugs were administered (*i.p*) immediately after envenomation at 0 hour.

### Sterile inflammatory markers

**Figure 2:**
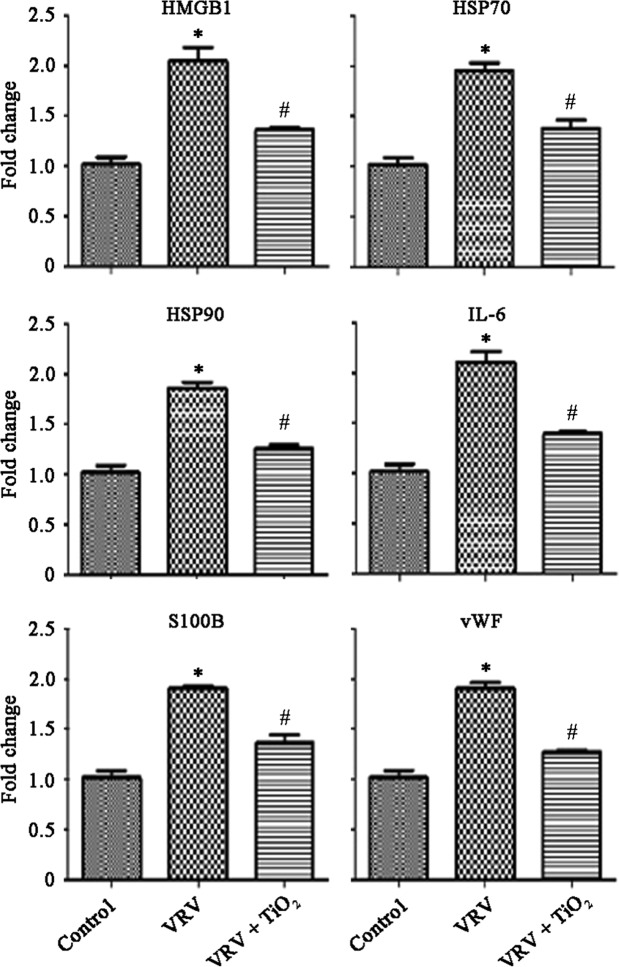
Viper (*Daboia russelii*) venom (VRV) induced inflammatory changes in experimental animals. Results expressed as mean ± SEM (n = 6). Results obtained are significantly different from control group (*P < 0.05) and *Daboia russelii* venom (^#^P < 0.05).

**Figure 3:**
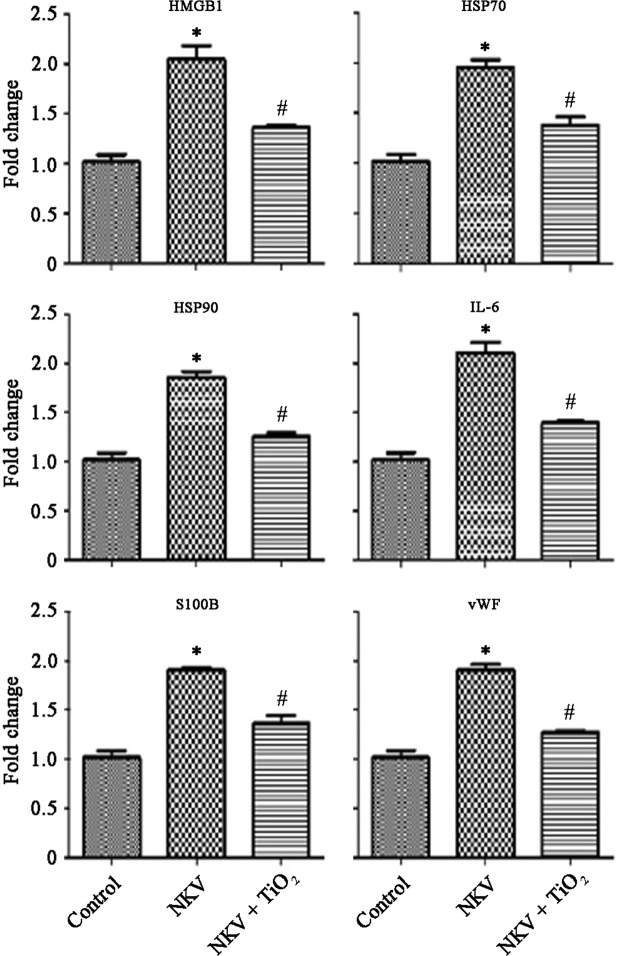
Cobra (*Naja kaouthia*) venom (NKV) induced inflammatory changes in experimental animals. Results expressed as mean ± SEM (n = 6). Results obtained are significantly different from control group (*P < 0.05) and *Naja kaouthia* venom (^#^P < 0.05).

The sterile inflammatory markers HMGB1, HSP70, HSP90, IL-6, S-100B and vWF were found to increase in Viper and Cobra venoms treated mouse while treatment with TiO_2_-NPs significantly reduced confirming the anti-inflammatory effects of TiO_2_-NPs (Figs 2 and 3).

## Discussion

**Figure 4:**
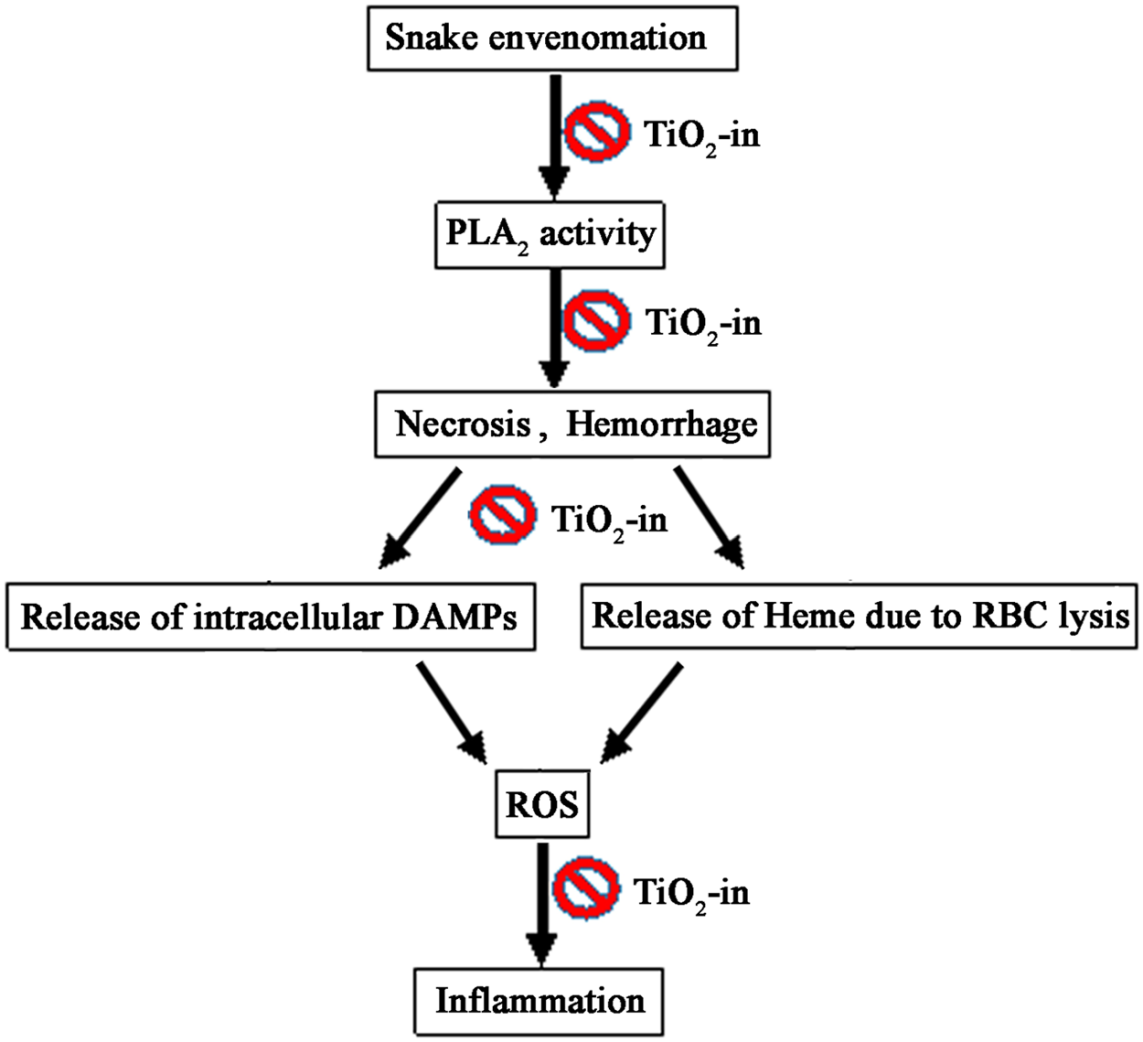
Possible mechanism of action of TiO_2_ [TiO_2_–in (inhibition)]. Snake venom-induced necrosis, haemorrhagic, lethal and inflammation and its neutralization by TiO_2_-NPs.

Injury and death due to snake bite is major health hazards in tropical and subtropical countries. In India more than 250 species of snakes are found, out of which 50 are venomous. Cobra and viper are the common snakes found throughout India^32–34^. The majority of death occurs due to the bites of these snakes. Snake envenomation is characterized by severe damage caused by the toxic action of venom components. The venom contains multiple components that induce haemorrhage, necrosis, inflammation, nephrotoxicity, cardiotoxicity, hematoxicity and often leads to death^35,36^. Viper and cobra venom contains major parts of Phospholipase A_2_ (PLA_2_) and metalloproteinase. Viper venom is a rich source of PLA_2_ enzyme that leads to many pathophysiological disturbances in the victims^34^. These components directly damage the micro-vessels, with consequent increase in haemorrhage and oedema^34^. The inflammatory effect induced by viper envenomation is due to the presence of PLA_2_ enzyme in venom^34–37^. The inflammatory effects induced by PLA_2_ in response to cobra and viper venoms are primarily related to the action of this enzyme on the membrane phospholipids and the release of eicosanoid precursors^25,38–42^. In present work, we characterized the oedematogenic, haemorrhagic, lethal and anti-inflammatory effects induced by the viper venom and its neutralization by TiO_2_-NPs (Fig. 4). Nanomedicine is a rapidly evolving field that provide enhanced diagnostic imaging and treatment of diseases known as theranostic. On account of improved health care, theranostic approaches are employed to make drug delivery more efficient and target specific. Theranostic nanomedicine can also be used for different purposes like non-invasive assessment of the pharmacokinetics, bio-distribution and the target site localization of conjugated or entrapped active agents^43,44^. Therefore TiO_2_-NPs is considered as a suitable target for medicine delivery as well as for detection of target cells due to its high fluorescence property under the excitation of normal white light in *in vitro* condition.

In this study, we have shown that TiO_2_-NPs are effective agent that may be used as a treatment in snake venom induced pathophysiological changes. TiO_2_ -NP arrays have been fabricated on p-type Si substrate using a cost effective (GLAD, PVD) technique. The surface topography revealed that the average size of TiO_2_-NPs is in nano-metric range. The element mapping of FESEM image indicates the presence of Titanium (Ti) and Oxygen (O) in the sample. The stability of TiO_2_-NPs synthethised by glancing angle deposition technique was calculated by its zeta potential. The continuous SAED analysis of TiO_2_-NPs reveals that particles are having crystalline behaviour with fine stability. So far, we have seen that TiO_2_-NPs effectively neutralize the viper and cobra venom-induced lethal activities both in *in vitro* and *in vivo* studies. TiO_2_-NPs also effectively neutralized the viper venom induced haemorrhagic activity in experimental animals. The fold of venom induced lethal and haemorrhagic activity was always found to be higher in *in vitro* than *in vivo* studies. The TiO_2_-NPs were also found to be more effective in viper venom induced pathophysiological changes than the cobra venom. The exact mechanism of action is still unclear. Among the several, one of the consequences of snake envenomation is increased DAMP molecules. Here, we have shown for the first time that the sterile inflammatory markers like IL-6, HSP, vWF, S100B and HMGB1 were increased by the administration of both cobra and viper venoms in male albino mice. The venoms may act through the stimulation of arachidonic acid pathway that leads to the generation of ROS and cytokines which leads to haemorrhage and tissue injury^41,45,46^. Tissue injury caused due to snake envenomation could be because of different mechanism. One of the mechanisms is inflammatory response which entails the generation of Hsp70, Hsp90, HMGB1, IL6, vWF and S100b. They are all generated through the arachidonic acid pathway. The metabolism of arachidonic acid by lipo-oxygenase or cyclo-oxygenase causes the release of pro-inflammatory marker proteins. Presence of PLA_2_ in viper venom helps in the conversion of the phospholipids to arachidonic acid which causes release of cytokines and leukotrienes^25,47,48^. The TiO_2_-NPs inhibits the production of sterile inflammatory proteins in venom-induced experimental rodents. The fold of protection is higher in case of viper venom than the cobra venom may be because of the difference in PLA_2_ concentration. PLA_2_ is higher in viper venom than the cobra venom. Further study may provide new biological probes in the treatments of snakebite. TiO_2_-NPs have shown better results in *in vitro* than in *in vivo* experiments. Our present investigation showed that the TiO_2_-NPs is effective against venom-induced pathophysiological conditions and could be taken as a potential antidote. The present work may be helpful to treat victims of snakebite especially in rural parts of India where availability of antiserum is limited. We are engage to explore the mechanism of venom inhibition by TiO_2_-NPs as a potential therapeutic agent against snake envenomation^49,50^.
